# Small Prokaryotic DNA-Binding Proteins Protect Genome Integrity throughout the Life Cycle

**DOI:** 10.3390/ijms23074008

**Published:** 2022-04-04

**Authors:** Katja Molan, Darja Žgur Bertok

**Affiliations:** 1 Department of Microbiology, Biotechnical Faculty, University of Ljubljana, 1000 Ljubljana, Slovenia; katja.molan@bf.uni-lj.si; 2 Department of Biology, Biotechnical Faculty, University of Ljubljana, 1000 Ljubljana, Slovenia

**Keywords:** DNA protection, nucleoid-associated proteins, small acid soluble proteins, single-stranded DNA-binding proteins

## Abstract

Genomes of all organisms are persistently threatened by endogenous and exogenous assaults. Bacterial mechanisms of genome maintenance must provide protection throughout the physiologically distinct phases of the life cycle. Spore-forming bacteria must also maintain genome integrity within the dormant endospore. The nucleoid-associated proteins (NAPs) influence nucleoid organization and may alter DNA topology to protect DNA or to alter gene expression patterns. NAPs are characteristically multifunctional; nevertheless, Dps, HU and CbpA are most strongly associated with DNA protection. Archaea display great variety in genome organization and many inhabit extreme environments. As of yet, only MC1, an archaeal NAP, has been shown to protect DNA against thermal denaturation and radiolysis. ssDNA are intermediates in vital cellular processes, such as DNA replication and recombination. Single-stranded binding proteins (SSBs) prevent the formation of secondary structures but also protect the hypersensitive ssDNA against chemical and nuclease degradation. Ionizing radiation upregulates SSBs in the extremophile *Deinococcus radiodurans*.

## 1. Introduction

All organisms must ensure accurate replication and maintenance of their genomes. Furthermore, bacteria must quickly adapt to a changing and often stressful environment. Coordinated responses by bacterial global regulatory systems enable their survival and adaptation [1]. In bacteria, mechanisms of genome maintenance must provide protection throughout the physiologically distinct phases of the life cycle, while spore formers must also maintain genome integrity within the dormant endospore. 

Genome integrity of all organisms is persistently threatened by endogenous and exogenous assaults. Exogenous DNA-damaging agents are physical (UV and ionizing irradiation, oxidants and drugs) as well as chemical (oxidizing, crosslinking and alkylating) and are encountered in the natural environments, including host defense mechanisms targeting pathogens. Endogenous agents are the result of cellular metabolism, such as reactive oxygen species (ROS), stalled replication forks and defects following recombination or chromosome segregation [2,3]. To cope with stress and DNA damage, bacteria have evolved global regulatory systems, DNA damage repair as well as DNA protective mechanisms. 

DNA repair and DNA protection mechanisms are ubiquitous. In addition, a number of bacteria have evolved a coordinated response to DNA damage controlling high fidelity repair mechanisms, as well as low fidelity, damage tolerance. In *Escherichia coli*, the inducible DNA repair pathway is designated the SOS response and is controlled by two regulators, LexA, a repressor, and RecA, an inducer [4,5]. Following DNA damage and upon DNA replication, DNA polymerase stalls at a DNA lesion while helicase continues unwinding DNA, generating an increase in single stranded DNA (ssDNA). RecA is activated (RecA*) by binding to single stranded DNA forming a nucleoprotein filament that stimulates self-cleavage of LexA. In *E. coli*, LexA cleavage results in derepression of more than 50 SOS genes mostly involved in DNA repair and damage tolerance. Additional physical and chemical strategies must be employed to protect genome integrity in all physiological states. These strategies mostly involve small DNA-binding proteins. Extremely resistant endospores are formed by spore-forming bacteria upon exposure to extreme environmental conditions that pose a threat to cell structures, including the genome. Small acid soluble DNA-binding proteins (SASPs) play a crucial role in genome protection within endospores [6]. In addition, hypersensitive single stranded DNA (ssDNA) intermediates are generated during vital processes, such as DNA replication, recombination and repair [7]. The single stranded binding proteins (SSBs), while playing a broader role in genome maintenance, also protect ssDNA. 

**Figure 1:**
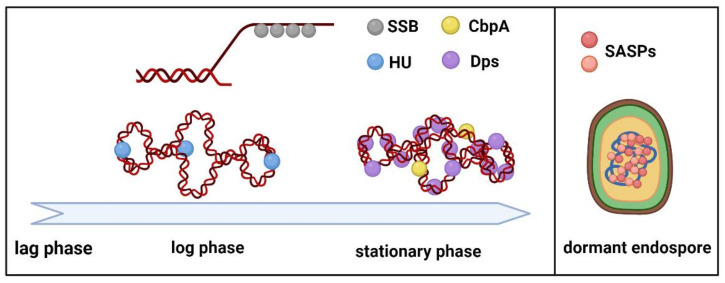
DNA-binding proteins that protect DNA through the bacterial growth cycle. Among spore-forming bacteria SASPs protect genomes in dormant endospores.

Here we focus on physical and chemical DNA protection provided by several well characterized small bacterial DNA-binding proteins, namely, nucleoid-associated proteins (NAPs), SASPs and SSBs (Figure 1). Archaeal NAPs are also discussed.

## 2. Bacterial Nucleoid-Associated Proteins and Genome Protection

While eukaryotes harbor histones that are responsible for packaging DNA into nucleosomes, all bacterial species possess abundant small proteins designated nucleoid-associated proteins (NAPs). NAPs are small basic proteins with dimerization/oligomerization domains that promote chromosome binding to compact, structure and regulate large portions of bacterial chromosomes. NAPs generally exhibit low sequence specificity; however, they may exhibit a preference for AT-rich sequences and/or specific DNA structures. [8,9,10]. The main *E. coli* NAPs are HU (heat-unstable protein), FIS (factor of inversion stimulation, H-NS (histone-like nucleoid structuring), IHF (integration host factor) and Dps (DNA-binding protein from starved cells). 

The expression patterns and intracellular concentrations of NAPs vary with regard to cell physiology. Thus, HU, IHF, Fis and H-NS are the primary growth phase NAPs with concentrations dropping as cells enter stationary phase while Dps levels peak in stationary phase/starving cells. In addition, activity of NAPs may be regulated by post-translational modification [11,12,13].

Characteristic of most bacterial protein DNA protecting mechanisms is non-specific DNA binding, possibly in combination with specific stronger binding, that jointly confer nucleoid protection and flexibility. NAPs in general play a crucial role in a rapid bacterial response to stress; nonetheless, two extensively studied, HU and Dps, and one as of yet less investigated, CbpA, exhibit the most pronounced DNA protection activity. The HU protein also plays significant roles in regulating global gene expression while the Dps protein is predominantly involved in DNA protection. 

### 2.1. HU

HU is a multifunctional protein involved in DNA organization, condensation, replication, recombination, global gene expression, including virulence, shape modulation and DNA protection [12,13]. In bacteria, HU is an abundant protein and the most conserved of the NAPs, binding throughout the bacterial genome. A typical *E. coli* cell harbors from 30,000 to 55,000 HU molecules [11,12,13], with concentrations highest during the exponential growth phase. HU has been shown to confer protection against a number of damaging agents: thermal denaturation [14], γ [15] and UV radiation [16]. HU also protects against oxidative stress conditions when hydroxyl radicals are generated. Further, HU has been shown to protect against exonuclease III, an intracellular nuclease [17]. In pathogenic *Helicobacter pylori*, the Hup protein (a homolog of *E. coli* HU), also showed protective activity to oxidative and acid stress as well as increased survival inside macrophages [18]. In addition to DNA protection, the HU protein has been shown to displace the LexA repressor, indicating that HU could assist in induction of transcription of the DNA damage inducible SOS genes [19]. 

In *E. coli*, two genes, *hupA* and *hupB*, encode subunits HUα and HUβ, respectively. In most bacteria, HU exists as a homodimer, while in *Enterobacteriacea,* HU also forms a heterodimer with subunits HU-α and HU-β [20]. In *E. coli,* HU dimers are formed by either HUα self-association (HUαα) or HUα-HUβ (HUαβ) interactions. HUα is mostly expressed during the early exponential growth phase while HUβ is expressed only during the stationary phase [21], indicating distinct roles of HUαα/DNA and HUαβ/DNA nucleoid structuring during growth and quiescence. HUα2 homodimers are predominant in the early log phase while HUαβ heterodimers mainly in the late log and stationary phase [22]. In *Mycobacterium tuberculosis*, HU forms a HU-β homodimer [23], whereas *Clostridium difficile* harbors only a *hupA* gene [24]. 

The HU protein is characterized by a 90 amino acid central region, with variations among protein homologs from different species mostly in the N- and C-terminal extensions [25,26,27]. The structures of HU alone and HU–DNA complexes have been determined using X-ray crystallography and NMR [25,26,28]. The protein has an “arm” consisting of two β-sheets extended to two β-ribbon parts and an α helical part (“the body”) [29]. Two HU DNA binding modes have been described: a DNA bending mode [25,26] and an extension mode with HU filaments forming on DNA [29,30]. 

HU binds weakly and nonspecifically to bulk chromosomal double stranded (dsDNA) DNA. However, HU displays a preference for AT-rich sequences and high affinity binding, in the nanomolar range, for specific or damaged DNA structures, such as four-way junctions, gaps, or nicks [17]. The high concentration of HU molecules per cell indicate that the majority of interactions are sequence independent.

A recent study employing single molecule tracking, revealed that HU exhibits nonspecific, weak, and transitory interactions with chromosomal DNA. Three conserved, surface-exposed lysine residues, previously shown to be responsible for nonspecific binding to DNA [12] were found to be crucial for these interactions. On the other hand, a conserved proline residue (P63) and the HUβ subunit could promote nucleoid compacting via binding to a specific DNA structure [31]. The authors proposed that HU, due to its differential interactions with chromosomal DNA, plays a dual role in maintaining proper nucleoid volume. While HU compacts the nucleoid through specific DNA structure-binding interactions, it decondenses the nucleoid through many nonspecific, weak and transitory interactions with the bulk chromosome. Such dynamic interactions may contribute to the viscoelastic properties and fluidity of the bacterial nucleoid required to facilitate proper chromosome function. 

*Deinococcus radiodurans* is an extremophilic organism that is highly resistant to radiation (e.g., ionizing radiation, UV light) [32]. Due to its extreme tolerance to DNA damaging agents it is also a model organism for studies of anti-oxidation and DNA repair. In *D. radiodurans,* HU is an essential protein. It is a major NAP and is responsible for most of the nucleoid compaction [33]. Recently, experiments employing spinning-disk time-lapse microscopy and super-resolution imaging, revealed that *D. radiodurans* nucleoids are highly condensed but nonetheless dynamic, adopting multiple distinct configurations as the bacteria progress through the cell cycle. Studies of the dynamics of the highly abundant HU protein showed that it binds only loosely to DNA. *D. radiodurans* nucleoids were found to exhibit pronounced plasticity as cells progress through the cell cycle. The authors proposed that the characteristic loose binding of HU could facilitate the dynamic nature of the *D. radiodurans* nucleoids [34].

#### 2.1.1. HU Post-Translational Modifications 

Post-translational protein modifications are an integral part of intracellular signaling in cellular metabolic processes. HupB, the HU homolog of the human pathogen *M. tuberculosis*, was initially found to be phosphorylated on serine and threonine residues. It was proposed that HupB acts as a signal molecule that could be involved in the detection of exogenous cues and subsequently in adaptive changes essential for survival. Phosphorylation was shown to inhibit DNA binding activity while Thr65 and Thr74 were determined as necessary for the DNA-binding capacity of HupB [35]. Subsequently, other modifications of the HupB protein were demonstrated, namely acetylation at various lysine residues that also alters the ability for DNA binding and DNA compaction. Thus, in *M. tuberculosis*, acetylation leads to a decrease in its affinity for DNA [36]. To regulate HU binding, a deacetylation mechanism for acetylated HU is also present in *Mycobacterium* [37]. HU is also subject to methylation (Lys3, 86, 94, and 103 and Arg53, 54, and 55) [38]. In the *E. coli*, Huα has been shown to be succinylated at Lys86 [39].

#### 2.1.2. HU and Biofilms

A recent study has revealed that the HU protein is also involved in bacterial cell protection or rather bacterial community protection. Biofilms are structured bacterial communities attached to inert or living surfaces that create a protective environment for bacterial cells, enabling the survival of physical and chemical treatments including high-dose antibiotics [40]. The *E. coli* HU protein has been shown to act as a molecular glue attaching bacteria to extracellular DNA in biofilms [41]. 

Interestingly, while the HU protein was shown to displace the SOS LexA repressor that could induce the SOS DNA damage repair system, the SOS response has been shown to play a significant role in biofilm formation. HU could thus play a dual role in biofilm formation. 

### 2.2. Dps

In the natural environment bacterial growth is characterized by a “feast or famine lifestyle”: long periods of nutritional deprivation and only short periods of nutrient abundance that allow rapid growth [42]. 

Dps was initially characterized as an abundant protein in starved *E. coli* cells [43] with highest concentrations, 85,000–180,000 molecules, in the stationary phase and only 6000–8500 monomers in exponentially growing cells [44]. The Dps proteins belong to the ferritin family and play an important role in DNA protection and ROS detoxification, as well as iron uptake and storage. 

Dps homologs have been identified in more than 1000 species of bacteria and archaea [45]. Reduced survival of *dps* mutants under stress conditions showed that Dps provides protection against oxidative stress, starvation, UV and γ radiation, metal ion toxicity, thermal stress and acid stress [46,47]. Starvation and oxidative stress have been shown to provoke the most pronounced Dps-mediated rearrangements in bacterial DNA, observed as tightly packed toroid-like or even crystal-like structures. Thus, Dps confers two types of protective activities; the first, the chemical—oxidation of Fe^2+^ ions with accumulation of the formed Fe_2_O_3_ within the Dps protein cavity, and the other, physical—DNA binding and genome condensation [48]. 

**Figure 2:**
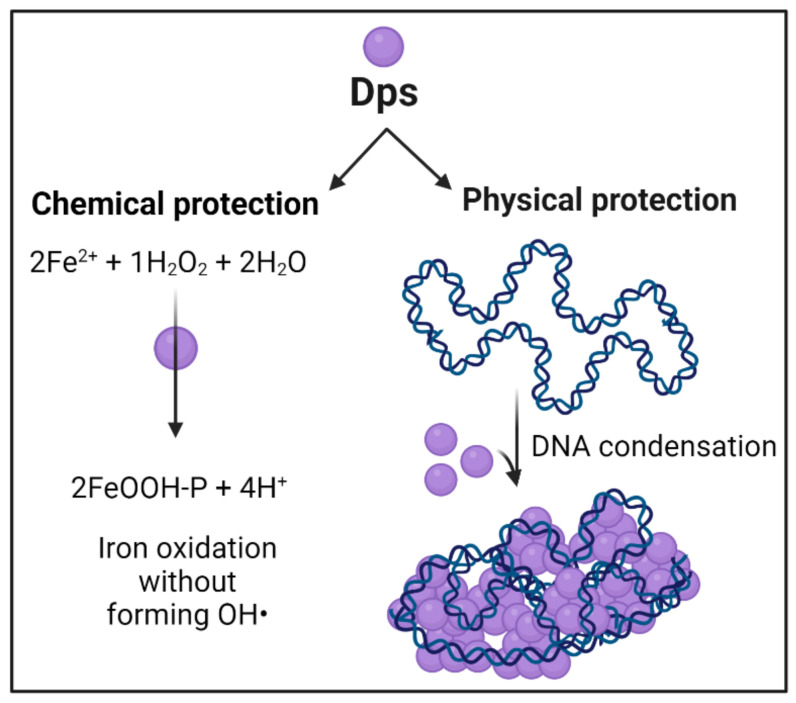
Dps protects DNA via chemical and physical activities.

Although iron is essential for many biological processes, free ferrous ions are toxic. The oxidation of iron in a bacterial cell occurs as a result of the Fenton reaction with the formation of a hydroxyl radical. The latter provoke peroxidation of lipids, DNA damage, and degradation of biomolecules. The Dps protein oxidizes iron in its ferroxidase center without forming hydroxyl radicals, protecting cell structures [49]. Further, Fe^3+^ ions remain bound to the protein, forming an ionic core inside the Dps molecule, harboring several hundred ions. The ferroxidase activity of Dps is similar to that of other ferritins; however, Dps preferentially oxidizes ferrous iron using hydrogen peroxide. Further, in Dps, 12 catalytic centers are formed by surfaces of 2 adjacent subunits, rather than being located within the 4-helix bundle of each subunit. The sequestered iron within the Dps protein cavity can be released by reduction. Dps iron oxidation and DNA binding are thus two separate functions that occur independently [48]. Jointly, they ensure cell viability by providing significant genome protection (Figure 2).

Proteins of the Dps family are composed of 12 identical or similar subunits, and form spherical particles with an internal cavity of ~4.5 nm. In *E. coli* Dps is a homododecamer with 2–3 tetrahedral symmetry. Each subunit contains 167 amino acids and has a molecular weight of 18.7 kDa [50]. Purified *E. coli* Dps molecules have been shown to self-aggregate in solution and upon addition of DNA, Dps dodecamers undergo extensive aggregation to rapidly form multi-layered plate-like crystals [51]. Co-crystallization of DNA and Dps is a defense strategy as DNA sequestration is a highly efficient means of protection against an array of environmental assaults. Iron ions stabilize the dodecameric form of Dps [51,52]. In *E. coli*, the N-terminal tails with positively charged lysine residues are responsible for interaction with DNA, self-aggregation and DNA condensation. Dps interacts with the bacterial chromosome/DNA via twelve unstructured N-terminal tails containing three lysine and one arginine residues [53]. Dps subunits form small pores that connect the internal cavity with solvent and can be used for the passage of ions and small molecules [54]. In vitro, the ability of Dps to choose one of two competing DNA fragments for complex formation was demonstrated, indicating that Dps may exhibit some sequence or structural selectivity. Atomic force microscopy demonstrated some preference of Dps for the ends of linear DNA fragments and even higher affinity for the branching point of artificial three-way junction molecules [54]. A recent study showed that in DNA–Dps complexes, DNA does not wrap Dps molecules and that a Dps molecule contacts with a DNA segment ~6 nm in length. The authors propose that DNA could be arranged along the rows of ordered protein molecules in a sheet of a Dps crystal [55]. 

Condensation protects the genome from various assaults. As stress is mitigated, it is believed that genome de-condensation should occur. Cell components whose presence is associated with cell physiology could modulate Dps–DNA interactions. Two metabolites, d-glucoronate and d-galacturonate, have been shown to provoke the dissociation of Dps dodecamers [56]. Further, Mg^2+^ ions (MgCl_2_) block the interaction of the Dps N-terminals with DNA, while the addition of Fe^2+^ (FeSO_4_) provokes complex destruction and aggregation as well as the formation of iron-containing clusters in the central cavity next to the acidic pore [57]. 

Bacteria mostly harbor one or two Dps proteins. While the amino acid residues responsible for Fe^2+^ binding are identical in all DPS proteins, differences are observed in their DNA binding activity and DNA condensation [58,59,60,61,62,63]. Interestingly, while most bacteria produce one or two Dps enzymes, the cyanobacterium *Nostoc punctiforme* produces five Dps proteins (NpDps1–5). Studies have indicated physiological differences among the five proteins, as well as cell-specific expression [64].

Dps is associated with physical and chemical DNA protection; however, other NAPs, namely, H-NS, Fis, IHF as well as HU, play significant roles in regulating bacterial gene expression. Nonetheless, investigation of potential Dps structural or sequence preferences employing ChIP-seq revealed target sites that appeared enriched with inverted repeats and overlapped with the binding sites of several other bacterial nucleoid proteins. The authors suggested that Dps could modulate transcription of at least those genes that are regulated by transcription factors that bind DNA targets with a lower affinity than Dps [65]. 

Dps–DNA complexes condense and protect DNA to overcome stress; nevertheless, DNA regions must be accessible to the transcription machinery. A recent study showed that deletion of *dps* decompacted the nucleoid but did not affect the transcriptome [66]. Single-molecule assays demonstrated that Dps dynamically condensed DNA around elongating RNAP without impeding its activity. The authors proposed that, rather than forming static crystalline structures, Dps forms dynamic complexes with diffusive properties similar to liquid–liquid phase separated organelles [67]. While RNAP can freely enter these organelles from the cytoplasm, other proteins, such as the tested restriction endonucleases, cannot. Differential solubility of macromolecules in Dps–DNA complexes could provide a simple mechanism for Dps protection that nonetheless allows transcription to proceed under stress. 

A recent investigation [68] employing electron microscopy, electron tomography, and EDX revealed in dormant *E. coli*, three morphologically distinct types of Dps promoted DNA condensation: nanocrystalline [68,69], liquid crystalline [70] and a novel, folded nucleosome-like. Thus, upon Dps-promoted condensation, DNA does not form a unique compact structure but may consist of various regions, each with a characteristic degree of internal order. Multiple types of DNA condensation in a dormant *E. coli* cell should enhance survival and enable rapid resumption of growth when returned to favorable/growth-proficient conditions. 

Dps ferroxidase activity and bacterial DNA condensation in stress conditions are its most significant roles; nonetheless, Dps is also involved in other cellular processes. Thus, Dps family proteins also participate in biofilm formation [71] and have been found among components of the outer membrane [72]. 

### 2.3. CbpA

Another NAP that protects DNA, albeit less intensively investigated, is the *E.* coli-curved DNA-binding protein A (CbpA). As its name indicates, CbpA was initially isolated as a protein binding with high affinity to intrinsically curved, AT-rich, DNA [73,74]. CbpA is produced in the stationary phase upon starvation, and protects DNA from damage by forming DNA aggregates morphologically similar to those formed by Dps. CbpA exists as monomers or dimers and is present in lower levels than Dps, and stationary phase cells accumulate between 3000 and 15,000 molecules [75]. In addition to DNA binding, CbpA acts as a co-chaperone [76]. The CbpA protein is highly conserved in many γ-proteobacteria (Table 1).

CbpA exhibits a three-domain structure: the N-terminal domain, a flexible linker and two C-terminal domains (CTDI and CTDII). The N-terminal J-domain is highly conserved among DnaJ-like co-chaperones that interact with DnaK, a chaperone and CbpM, an inhibitor of CbpA. The linker-CTDI region is responsible for DNA binding, while the CTDII region mediates dimerization, required for interactions with DNA. CpbA interacts with the DNA minor groove and a highly conserved arginine residue is required [77]. 

## 3. Archaeal NAPs

Archaea represent the third domain of life, along with eukarya and bacteria. They are morphologically similar to bacteria; however, there are notable differences in the lipid component of the cell membrane, the structure of the replication machinery as well as the RNA polymerase [78]. Many archaea grow in extreme conditions, such as high temperatures (thermophiles) that would denature purified DNA, at high salt concentrations (halophiles) and at acidic pH (acidophiles) [79]. 

Chromosome dynamics/organization and protection is less explored among archaea. Nonetheless, studies have revealed greater variability as archaea employ NAPs and/or histones. On the basis of proteins involved in chromosome organization, archaea may be divided into two groups: (i) the majority of species in the phylum *Euryarchaeota* encode proteins homologous to histones, while (ii) those in *Crenarchaeota* employ other DNA binding proteins such as Alba, Cren7 and Sul7d [80,81,82]. Thus, the crenarchaea nucleoid exhibits similarities with that of bacteria. 

Of the archaeal DNA-binding proteins, Alba and histones are the most widely distributed. Alba is a 10 kDa DNA/RNA-binding protein that forms a dimer and has a mixed α-helix and β-strand structure. The protein undergoes post-translational modifications and acetylation lowers binding affinity. The Alba proteins are present in all archaea with the exception of two classes of *Euryarchaea*, *Methanomicrobia* and *Haloarchaea* [82]. They exhibit a variety of functions: genome packaging and organization, regulate transcription, RNA metabolism as well as processes involved in development and differentiation. Archaea without Alba, harbor the Methanogen Chromosomal protein 1 (MC1). DNA bound to MC1 has been shown to be protected against thermal denaturation and radiolysis. MC1 compacts DNA as a monomer inducing a V-turn on DNA [83]. Among *Euryarchaeota* that lack histones are species in *Thermoplasmatales*. They encode HTa, a protein homologous to the bacterial HU [84]. HTa is associated with genomic DNA and protects a minimum of about 40 bp. Phylogenetic analysis indicates that HTa was horizontally transferred from bacteria to archaea. Several recent studies on HTa have shown that, in contrast to bacterial HU, HTa in *Thermoplasma* wraps DNA, forming particles of approximately 6 nm [84]. In *Euryarchaeaota*, DNA wrapping seems to be required for DNA folding, regardless of the protein used (histone or HTa) (Table 2). Nevertheless, a protective role of HTa as observed for HU in bacteria, has not been investigated. 

Ferritins are highly conserved proteins that are widely distributed from prokaryotes to humans. Prokaryotes in general possess three types of ferritin: typical ferritin, bacterioferritin, and Dps. A study investigating ferritin genes among 248 genomes of various species, demonstrated considerable numbers of ferritin genes, including genes for the Dps protein among archaea [85].

Extreme environments represent challenges for organizing, structuring and protecting DNA. Damage repair and protective mechanisms against osmotic stress and intense UV radiation among halophilic archaea [86], as well as repair systems in hyperthermophiles, have been studied. Surprisingly, no unique DNA repair systems have been discovered. It has been suggested that crosstalk of archaeal DNA repair pathways, their close links with replisome components [87], and perhaps additional not yet discovered mechanisms, successfully protect their genomes.

## 4. Small Acid Soluble DNA Binding Proteins

To survive in adverse environmental conditions, such as lack of essential nutrients and moisture, exposure to toxic chemicals, radiation or high temperatures, the vegetative bacteria of a few genera, notably *Bacillus* and *Clostridium,* can undergo differentiation to produce an endospore. 

Endospores are dormant forms of bacteria that withstand extreme environmental conditions and chemical exposures [88]. The combined activity of several spore components contribute to their pronounced resistance: (i) outer coat layers and associated pigments protect against UV radiation and chemicals; (ii) the peptidoglycan cortex; (iii) a relatively impermeable inner spore membrane; (iv) saturation of DNA with α/β-type small, acid-soluble spore proteins that alter DNA structure and result in protection against UV and γ-radiation, genotoxic chemicals, and wet or dry heat; (v) high levels of Ca^2+^ with dipicolinic acid (CaDPA) located in the spore core; and (vi) DNA repair enzymes in the spore core, including spore specific, which repair spore DNA damage and are active upon germination and outgrowth [89,90]. 

Spore formation and resistance have been extensively studied in *Bacillus subtilis*. Additionally, spores of a number of species are vectors of food spoilage and food-borne disease (*Clostridium botulinum*, *Clostridium perfringens*), involved in human diseases in hospitals and long-term nursing facilities (*C. difficile*) [91] and potential biological weapons (*Bacillus anthracis*) [92,93]. Knowledge on resistance properties and resistance mechanisms is essential for the development of efficient prevention and interventions. Sporulation is initiated by activation of the key regulator Spo0A through the phosphorelay in response to environmental cues/stress [94]. A cascade of sigma factor activation and sporulation specific gene expression follows, ultimately leading to development of a dormant and resistant endospore [95]. 

A key factor in spore resistance are the small acid-soluble proteins (SASPs) that are produced by all endospore forming organisms. Studies employing mutants lacking most SASPs have demonstrated that they are most significant in protecting spore DNA from UV and γ radiation, but also in protecting against desiccation, wet and dry heat, toxic chemicals and enzymes [96]. The SASPs are 60 to 75 a.a. in length, and are highly conserved among endospore-forming organisms. They are produced late in sporulation under the forespore-specific sigma factor, σ^G^, and comprise up to 20% of the total spore proteins. Most spore-forming bacteria harbor two major α/β-type SASPs (SspA and SspB), encoded by multiple monocistronic genes. α/β-type SASPs bind in the DNA minor groove. Spore-forming bacteria produce other minor SASPs that vary in number depending on the organism. Thus, in *B. subtilis*, a γ-type SASP serves as an amino acid reservoir required during outgrowth from the germinated spore [96,97]. DNA in spores is saturated with SASPs that bind nonspecifically and their affinity for DNA is not extremely high. Nevertheless, due to the low water content of the spore core, the SASPs remain tightly bound to DNA. Upon germination and outgrowth, the α/β-type SASPs dissociate from DNA and are degraded by a specific endonuclease. 

DNA binding of the α/β-type SASPs provokes a change in DNA UV photochemistry. It is well known that UV irradiation of DNA generates photoproducts, cyclobutene dimers (CDPs), and 6-4 adducts (64PP). In spores, UV radiation generates a spore photoproduct (SP) that is less lethal due to relatively error-free repair. Saturation of DNA with α/β-type SASPs changes DNA conformation from a B to an intermediate structure (between A and B DNA) altering DNA photochemistry. Both low water content and high levels of DPA also contribute to the altered DNA photochemistry [96,97,98]. 

The structure of the SASP–DNA complex also provides protection against other stressors. Thus, exposure to dry and/or wet heat results in cleavage of the glycosylic bond. The α/β SASP–DNA complex impedes cleavage of the glycosylic bond between a deoxyribose and a base due to tighter packing than in B-DBA, occlusion of the sugar–phosphate backbone, and alteration of the bond angle from the deoxyribose to the base. The α/β-type SASP–DNA structure is also significant for protection against DNA damage by toxic chemicals, such as nitrous acid and formaldehyde, due to restricted access. In DNA, the imino/amino groups of guanine are in the minor groove, and are in vitro shielded due to interaction with SASP amino acid residues. SASPs are well-conserved across genera [96,98,99]. 

All SASPs protect spore DNA; however, some variation with regard to their roles is observed. In *B. subtilis*, the α/β-type SASPs contribute to spore resistance against a number of chemicals, such as nitrous acid, formaldehyde, glutaraldehyde, iodine, or hydrogen peroxide [98,99] while in *C*. *botulinum*, SASPs are not necessary for protection against hydrogen peroxide or formaldehyde [100]. In *C. perfringens* isolates that cause food poisoning, Ssp4 ensure spore protection against food processing procedures (e.g., high heat and use of nitrites). Other *C. perfringens* SASPs protect spores against UV light, hydrogen peroxide, nitrous acid, formaldehyde and hydrochloric acid [101,102,103,104,105]. In *C. difficile*, SspA predominately confers resistance to UV. Additionally, some of the SASPs provide only a minor nitrous acid spore resistance, but seemingly not to other chemicals. Surprisingly, deletion of both *sspA* and *sspB* prevented spore formation, indicating that the major *C. difficile* SASPs may bind specific DNA sequences to regulate spore formation/SASPs, and are also involved in spore formation [106].

## 5. Bacterial Single-Stranded DNA Binding Proteins

Single-stranded DNA-binding proteins (SSBs) are essential in all living organisms. They interact/bind with ssDNA with high affinity in a sequence independent manner. DNA replication, recombination and repair require unwinding of double-stranded genomic DNA to generate ssDNA intermediates. SSBs bind to prevent the formation of secondary structures and protect the hypersensitive ssDNA from chemical as well as nuclease degradation [107]. Further, SSBs binding to DNA assists in the formation of nucleoprotein complexes required for DNA replication, repair and recombination [108] and, thus, in genome maintenance. All characterized SSB proteins harbor an oligonucleotide/oligosaccharide-binding (OB) fold responsible for ssDNA binding [109]. The OB fold consists of at least a five stranded beta-sheet arranged as a beta barrel capped by an alpha helix. Rapid diffusion of SSB along ssDNA seems to be conserved among different types of SSB proteins.

Although the majority of bacterial SSB proteins function as homotetramers [110], dimeric SSB proteins have been described among extremophiles, namely, of the genera *Deinococcus* and *Thermus* [111]. The dimeric SSB proteins from *D. radiodurans* and from *T. thermophilus* also harbor four OB folds, two per monomer. Interestingly, in response to ionizing radiation, SSB levels increase in *D. radiodurans* from ~20,000 to 56,000 dimers per cell, in comparison to *E. coli*, which maintains 200–3000 EcSSB tetramers regardless of DNA damaging treatment [112]. The large number of DraSSB dimers per cell and its rapid increase in response to ionizing radiation confer high tolerance of *D. radiodurans* to the tested DNA-damaging agents [113]. Nevertheless, their ssDNA-binding affinities are considerably lower [114]. Comparably to homotetrameric SSB proteins, the investigated homodimeric SSB proteins also diffuse rapidly along ssDNA [115].

## 6. Conclusions

To survive, all organisms harbor multiple mechanisms that jointly provide DNA repair and DNA protection throughout the bacterial life cycle as well as in dormant endospores. Most of the proteins involved in genome protection mechanisms are multifunctional. In bacteria, Dps provides two mechanisms of protection, HU is also a vital transcription regulator and possibly a signal molecule, CbpA, a co-chaperone, while SSBs also play a broad role in genome maintenance. Limiting the stationary phase abundant Dps to chemical and physical DNA protection could provide efficient DNA shielding in the absence of replication, which is required for a number of DNA repair mechanisms. Proficient DNA protection with small DNA binding proteins is observed in extremophiles such as *D. radiodurans*. To organize their genomes, archaea combine strategies employed by bacteria and eukaryota. Studies are focused on the structure and interactions of the proteins involved while less is currently known of their roles in DNA protection. Additional studies will most certainly identify novel NAPs and DNA binding proteins that protect the prokaryotic genome against damaging assaults, and resolve the mechanisms they utilize. 

## Figures and Tables

**Table 1:** Characteristics of bacterial DNA protective NAPs.

NAPs	Structure	Protection	DNA Binding	Function	Identified in
**HU**	α_2,_ β_2,_ αβ	physical	chromosomal dsDNA, non-specific, weak; binding to AT-rich and specific DNA structures	nucleoid compaction, maintaining proper nucleoid volume	bacteria
**Dps**	α_12_	physical, chemical	chromosome/DNA, some sequence or structural selectivity	iron oxidation in ferroxidase centre; DNA condensation, co-crystallization with DNA	in ˃ 1000 species of bacteria and archaea
**CbpA**	monomer or dimer	physical	AT-rich DNA, non-specific	forms DNA aggregates, co-chaperone	γ-proteobacteria, highly conserved

**Table 2:** Characteristics of archaeal NAPs.

NAPs	Structure	DNA Binding	Function	Identified in
**Alba**	dimer	DNA/RNA binding	genome packaging and organization	all archea except *Methanomicrobia, Haloarchaea*
**MC1**	monomer	non-specific binding; prefers bent DNA	compacts DNA induces a V-turn on DNA; protects against thermal denaturation and radiolysis	*Methanomicrobia, Haloarchaea*
**HTa** (HU homolog)	histone-like oligomerization	GC-rich DNA	wraps DNA, required for DNA folding	*Thermoplasmatales*

## Data Availability

All data are available in the manuscript.

## References

[1] Foster P.L. (2007). Stress-induced mutagenesis in bacteria. Crit. Rev. Biochem. Mol. Biol..

[2] Pennington J.M., Rosenberg S.M. (2007). Spontaneous DNA breakage in single living *Escherichia coli* cells. Nat. Genet..

[3] McGlynn P., Savery N.J., Dillingham M.S. (2012). The conflict between DNA replication and transcription. Mol. Microbiol..

[4] Butala M., Žgur-Bertok D., Busby S.J.W. (2009). The bacterial LexA transcriptional repressor. Cell. Mol. Life Sci..

[5] Maslowska K.H., Makiela-Dzbenska K., Fijalkowska I.J. (2019). The SOS system: A complex and tightly regulated response to DNA damage. Environ. Mol. Mutagen..

[6] Moeller R., Setlow P., Reitz G., Nicholson W.L. (2009). Roles of small, acid-soluble spore proteins and core water content in survival of *Bacillus subtilis* spores exposed to environmental solar UV radiation. Appl. Environ. Microbiol..

[7] Marceau A.H., Keck J. (2012). Functions of single-strand DNA-binding proteins in DNA replication, recombination, and repair. Single-Stranded DNA Binding Proteins.

[8] Dillon S., Dorman C. (2010). Bacterial nucleoid-associated proteins, nucleoid structure and gene expression. Nat. Rev. Microbiol..

[9] Verma S.C., Qian Z., Adhya S.L. (2019). Architecture of the *Escherichia coli* nucleoid. PLoS Genet..

[10] Hołówka J., Zakrzewska-Czerwińska J. (2020). Nucleoid associated proteins: The small organizers that help to cope with stress. Front. Microbiol..

[11] Azam T.A., Ishihama A. (1999). Twelve species of the nucleoid-associated protein from *Escherichia coli*. Sequence recognition specificity and DNA binding affinity. J. Biol. Chem..

[12] Azam T.A., Iwata A., Nishimura A., Ueda S., Ishihama A. (1999). Growth phase-dependent variation in protein composition of the *Escherichia coli* nucleoid. J. Bacteriol..

[13] Stojkova P., Spidlova P., Stulik J. (2019). Nucleoid-associated protein HU: A Lilliputian in gene regulation of bacterial virulence. Front. Cell. Infect. Microbiol..

[14] Rouvière-Yaniv J., Gros F., Haselkorn R., Reiss C. (1977). Histone-like Proteins in Prokaryotic Organisms and Their Interaction with DNA.

[15] Boubrik F., Rouviere-Yaniv J. (1995). Increased sensitivity to gamma irradiation in bacteria lacking protein HU. Proc. Natl. Acad. Sci. USA.

[16] Li S., Waters R. (1998). *Escherichia coli* strains lacking protein HU are UV sensitive due to a role for HU in homologous recombination. J. Bacteriol..

[17] Kamashev D., Rouviere-Yaniv J. (2000). The histone-like protein HU binds specifically to DNA recombination and repair intermediates. EMBO J..

[18] Wang G., Lo L.F., Maier R.J. (2012). A histone-like protein of *Helicobacter pylori* protects DNA from stress damage and aids host colonization. DNA Repair.

[19] Preobrajenskaya O., Boullard A., Boubrik F., Schnarr M., Rouvière-Yaniv J. (1994). The protein HU can displace the LexA repressor from its DNA-binding sites. Mol. Microbiol..

[20] Pettijohn D.E. (1988). Histone-like proteins and bacterial chromosome structure. J. Biol. Chem..

[21] Hammel M., Amlanjyoti D., Reyes F.E., Chen J.-H., Parpana R., Tang H.Y.H., Larabell C.A., Tainer J.A., Adhya S. (2016). HU multimerization shift controls nucleoid compaction. Sci. Adv..

[22] Claret L., Rouviere-Yaniv J. (1997). Variation in HU composition during growth of *Escherichia coli*: The heterodimer is required for long term survival. J. Mol. Biol..

[23] Bhowmick T., Ghosh S., Dixit K., Ganesan V., Ramagopal U.A., Dey D., Sarma S.P., Ramakumar S., Nagaraja V. (2014). Targeting *Mycobacterium tuberculosis* nucleoid-associated protein HU with structure-based inhibitors. Nat. Commun..

[24] Paiva A.M.O., Friggen A.H., Qin L., Douwes R., Dame R.T., Smits W.K. (2019). The bacterial chromatin protein HupA can remodel DNA and associates with the nucleoid in *Clostridium difficile*. J. Mol. Biol..

[25] Dame R.T. (2005). The role of nucleoid-associated proteins in the organization and compaction of bacterial chromatin. Mol. Microbiol..

[26] Dame R.T., Kalmykowa O.J., Grainger D.C. (2011). Chromosomal macrodomains and associated proteins: Implications for DNA organization and replication in gram negative bacteria. PLoS Genet..

[27] Dame R.T., Rashid F.-Z.M., Grainger D.C. (2019). Chromosome organization in bacteria: Mechanistic insights into genome structure and function. Nat. Rev. Genet..

[28] Vis H., Mariani M., Vorgias C.E., Wilson K.S., Kaptein R., Boelens R. (1995). Solution structure of the HU protein from *Bacillus stearothermophilus*. J. Mol. Biol..

[29] Swinger K.K., Lemberg K.M., Zhang Y., Rice P.A. (2003). Flexible DNA bending in HU-DNA cocrystal structures. EMBO J..

[30] Van Noort J., Verbrugge S., Goosen N., Dekker C., Dame R.T. (2004). Dual architectural roles of HU: Formation of flexible hinges and rigid filaments. Proc. Natl. Acad. Sci. USA.

[31] Bettridge K., Verma S., Weng X., Adhya S., Xiao J. (2021). Single-molecule tracking reveals that the nucleoid-associated protein HU plays a dual role in maintaining proper nucleoid volume through differential interactions with chromosomal DNA. Mol. Microbiol..

[32] Makarova K.S., Aravind L., Wolf Y.I., Tatusov R.L., Minton K.W., Koonin E.V., Daly M.J. (2001). Genome of the extremely radiation-resistant bacterium *Deinococcus radiodurans* viewed from the perspective of comparative genomics. Microbiol. Mol. Biol. Rev..

[33] Nguyen H.H., de la Tour C.B., Toueille M., Vannier F., Sommer S., Servant P. (2009). The essential histone-like protein HU plays a major role in *Deinococcus radiodurans* nucleoid compaction. Mol. Microbiol..

[34] Floc’H K., Lacroix F., Servant P., Wong Y.-S., Kleman J.-P., Bourgeois D., Timmins J. (2019). Cell morphology and nucleoid dynamics in dividing *Deinococcus radiodurans*. Nat. Commun..

[35] Gupta M., Sajid A., Sharma K., Ghosh S., Arora G., Singh R., Nagaraja V., Tandon V., Singh Y. (2014). HupB, a nucleoid-associated protein of *Mycobacterium tuberculosis*, is modified by serine/threonine protein kinases in vivo. J. Bacteriol..

[36] Ghosh S., Padmanabhan B., Anand C., Nagaraja V. (2016). Lysine acetylation of the *Mycobacterium tuberculosis* HU protein modulates its DNA binding and genome organization. Mol. Microbiol..

[37] Anand C., Garg R., Ghosh S., Nagaraja V. (2017). A Sir2 family protein Rv1151c deacetylates HU to alter its DNA binding mode in *Mycobacterium tuberculosis*. Biochem. Biophys. Res. Commun..

[38] Sakatos A., Babunovic G.H., Chase M.R., Dills A., Leszyk J., Rosebrock T., Bryson B., Fortune S.M. (2018). Posttranslational modification of a histone-like protein regulates phenotypic resistance to isoniazid in mycobacteria. Sci. Adv..

[39] Weinert B.T., Schölz C., Wagner S.A., Iesmantavicius V., Su D., Daniel J.A., Choudhary C. (2013). Lysine succinylation is a frequently occurring modification in prokaryotes and eukaryotes and extensively overlaps with acetylation. Cell Rep..

[40] Hall-Stoodley L., Costerton J.W., Stoodley P. (2004). Bacterial biofilms: From the natural environment to infectious diseases. Nat. Rev. Microbiol..

[41] Thakur B., Arora K., Gupta A., Guptasarma P. (2021). The DNA-binding protein HU is a molecular glue that attaches bacteria to extracellular DNA in biofilms. J. Biol. Chem..

[42] Kolter R., Siegele D.A., Tormo A. (1993). The stationary phase of the bacterial life cycle. Annu. Rev. Microbiol..

[43] Almiron M., Link A.J., Furlong D., Kolter R. (1992). A novel DNA-binding protein with regulatory and protective roles in starved *Escherichia coli*. Genes Dev..

[44] Talukder A.A., Ishihama A. (2014). Dps is a stationary phase-specific protein of *Escherichia coli* nucleoid. Adv. Microbiol..

[45] Calhoun L.N., Kwon Y.M. (2011). Structure, function and regulation of the DNA-binding protein Dps and its role in acid and oxidative stress resistance in *Escherichia coli*: A review. J. Appl. Microbiol..

[46] De la Garza-García J.A., Ouahrani-Bettache S., Lyonnais S., Ornelas-Eusebio E., Freddi L., Al Dahouk S., Occhialini A., Köhler S. (2021). Comparative genome-wide transcriptome analysis of *Brucella suis* and *Brucella microti* under acid stress at pH 4.5: Cold shock protein CspA and Dps are associated with acid resistance of *B. microti*. Front. Microbiol..

[47] Algu K., Choi V.S.C., Dhami R.S., Duncan D.A.K. (2007). Dps confers protection of DNA sequence integrity in UV irradiated *Escherichia coli*. J. Exp. Microbiol. Immunol..

[48] Karas V.O., Westerlaken I., Meyer A.S. (2015). The DNA-binding protein from starved cells (Dps) utilizes dual functions to defend cells against multiple stresses. J. Bacteriol..

[49] Grant R.A., Filman D., Finkel S.E., Kolter R., Hogle J.M. (1998). The crystal structure of Dps, a ferritin homolog that binds and protects DNA. Nat. Struct. Biol..

[50] Wolf S., Frenkiel D., Arad T., Finkel S.E., Kolter R., Minsky A. (1999). DNA protection by stress-induced biocrystallization. Nature.

[51] Antipov S., Turishchev S., Purtov Y., Shvyreva U., Sinelnikov A., Semov Y., Preobrazhenskaya E., Berezhnoy A., Shusharina N., Novolokina N. (2017). The oligomeric form of the *Escherichia coli* Dps protein depends on the availability of iron ions. Molecules.

[52] Ceci P., Cellai S., Falvo E., Rivetti C., Rossi G.L., Chiancone E. (2004). DNA condensation and self-aggregation of *Escherichia coli* Dps are coupled phenomena related to the properties of the N-terminus. Nucleic Acids Res..

[53] Chiancone E., Ceci P. (2010). The multifaceted capacity of Dps proteins to combat bacterial stress conditions: Detoxification of iron and hydrogen peroxide and DNA binding. Biochim. Biophys. Acta.

[54] Melekhov V.V., Shvyreva U.S., Timchenko A.A., Tutukina M., Preobrazhenskaya E.V., Burkova D.V., Artiukhov V.G., Ozoline O., Antipov S. (2015). Modes of *Escherichia coli* Dps interaction with DNA as revealed by atomic force microscopy. PLoS ONE.

[55] Dubrovin E.V., Dadinova L.A., Petoukhov M.V., Soshinskaya E.Y., Mozhaev A.A., Klinov D.V., Schäffer T.E., Shtykova E.V., Batishchev O.V. (2021). Spatial organization of Dps and DNA–Dps complexes. J. Mol. Biol..

[56] Bessonova T.A., Shumeiko S.A., Purtov Y.A., Antipov S.S., Preobrazhenskaya E.V., Tutukina M.N., Ozoline O.N. (2016). Hexuronates affect oligomeric form of a structural protein of bacterial nucleoid Dps and its ability to bind linear DNA fragments. Biophysics.

[57] Dadinova L., Kamyshinsky R., Chesnokov Y., Mozhaev A., Matveev V., Gruzinov A., Vasiliev A., Shtykova E. (2021). Structural rearrangement of Dps-DNA complex caused by divalent Mg and Fe cations. Int. J. Mol. Sci..

[58] Papinutto E., Dundon W.G., Pitulis N., Battistutta R., Montecucco C., Zanotti G. (2002). Structure of two iron-binding proteins from *Bacillus anthracis*. J. Biol. Chem..

[59] Ceci P., Mangiarotti L., Rivetti C., Chiancone E. (2007). The neutrophil-activating Dps protein of *Helicobacter pylori*, HP-NAP, adopts a mechanism different from *Escherichia coli* Dps to bind and condense DNA. Nucleic Acids Res..

[60] Roy S., Saraswathi R., Chatterji D., Vijayan M. (2008). Structural studies on the second *Mycobacterium smegmatis* Dps: Invariant and variable features of structure, assembly and function. J. Mol. Biol..

[61] Chowdhury R.P., Saraswathi R., Chatterji D. (2010). Mycobacterial stress regulation: The Dps “twin sister” defense mechanism and structure-function relationship. IUBMB Life.

[62] Ghatak P., Karmakar K., Kasetty S., Chatterji D. (2011). Unveiling the role of Dps in the organization of mycobacterial nucleoid. PLoS ONE.

[63] Santos S.P., Mitchell E.P., Franquelim H.G., Castanho M.A., Abreu I.A., Romão C.V. (2015). Dps from *Deinococcus radiodurans*: Oligomeric forms of Dps1 with distinct cellular functions and Dps2 involved in metal storage. FEBS J..

[64] Howe C., Ho F., Nenninger A., Raleiras P., Stensjö K. (2018). Differential biochemical properties of three canonical Dps proteins from the cyanobacterium *Nostoc punctiforme* suggest distinct cellular functions. J. Biol. Chem..

[65] Antipov S.S., Tutukina M.N., Preobrazhenskaya E.V., Kondrashov F.A., Patrushev M.V., Toshchakov S.V., Dominova I., Shvyreva U.S., Vrublevskaya V.V., Morenkov O.S. (2017). The nucleoid protein Dps binds genomic DNA of *Escherichia coli* in a non-random manner. PLoS ONE.

[66] Janissen R., Arens M.M.A., Vtyurina N.N., Rivai Z., Sunday N.D., Eslami-Mossallam B., Gritsenko A.A., Laan L., de Ridder D., Artsimovitch I. (2018). Global DNA compaction in stationary-phase bacteria does not affect transcription. Cell.

[67] Mitrea D.M., Kriwacki R.W. (2016). Phase separation in biology; functional organization of a higher order. Cell Commun. Signal..

[68] Loiko N., Danilova Y., Moiseenko A., Kovalenko V., Tereshkina K., Tutukina M., El-Registan G., Sokolova O., Krupyanskii Y. (2020). Morphological peculiarities of the DNA-protein complexes in starved *Escherichia coli* cells. PLoS ONE.

[69] Minsky A., Shimoni E., Frenkiel-Krispin D. (2002). Stress, order and survival. Nat. Rev. Mol. Cell Biol..

[70] Moiseenko A., Loiko N., Tereshkina K., Danilova Y., Kovalenko V., Chertkov O., Feofanov A.V., Krupyanskii Y.F., Sokolova O.S. (2019). Projection structures reveal the position of the DNA within DNA-Dps Co-crystals. Biochem. Biophys. Res. Commun..

[71] Theoret J.R., Cooper K.K., Zekarias B., Roland K.L., Law B.F., Curtiss R., Joens L.A. (2012). The *Campylobacter jejuni* Dps homologue is important for in vitro biofilm formation and cecal colonization of poultry and may serve as a protective antigen for vaccination. Clin. Vaccine Immunol..

[72] Lacqua A., Wanner O., Colangelo T., Martinotti M.G., Landini P. (2006). Emergence of biofilm-forming subpopulations upon exposure of *Escherichia coli* to environmental bacteriophages. Appl. Environ. Microbiol..

[73] Yamada H., Muramatsu S., Mizuno T. (1990). An *Escherichia coli* protein that preferentially binds to sharply curved DNA. J. Biochem..

[74] Meyer A.S., Grainger D.C. (2013). The *Escherichia coli* nucleoid in stationary phase. Adv. Appl. Microbiol..

[75] Bird J.G., Sharma S., Roshwalb S.C., Hoskins J.R., Wickner S. (2006). Functional analysis of CbpA, a DnaJ homolog and nucleoid-associated DNA-binding protein. J. Biol. Chem..

[76] Cosgriff S., Chintakayala K., Chim Y.T.A., Chen X., Allen S., Lovering A.L., Grainger D.C. (2010). Dimerization and DNA-dependent aggregation of the *Escherichia coli* nucleoid protein and chaperone CbpA. Mol. Microbiol..

[77] Chintakayala K., Sellars L.E., Singh S.S., Shahapure R., Westerlaken I., Meyer A.S., Dame R.T., Grainger D.C. (2015). DNA recognition by *Escherichia coli* CbpA protein requires a conserved arginine-minor-groove interaction. Nucleic Acids Res..

[78] Baker B.J., De Anda V., Seitz K.W., Dombrowski N., Santoro A.E., Lloyd K.G. (2020). Diversity, ecology and evolution of Archaea. Nat. Microbiol..

[79] Adam P.S., Borrel G., Brochier-Armanet C., Gribaldo S. (2017). The growing tree of Archaea: New perspectives on their diversity, evolution and ecology. ISME J..

[80] Peeters E., Driessen R.P., Werner F., Dame R.T. (2015). The interplay between nucleoid organization and transcription in archaeal genomes. Nat. Rev. Microbiol..

[81] Zhang Z., Zhao M., Chen Y., Wang L., Liu Q., Dong Y., Gong Y., Huang L. (2019). Architectural roles of Cren7 in folding crenarchaeal chromatin filament. Mol. Microbiol..

[82] Laursen S.P., Bowerman S., Luger K. (2021). Archaea: The final frontier of chromatin. J. Mol. Biol..

[83] Loth K., Largillière J., Coste F., Culard F., Landon C., Castaing B., Delmas A.F., Paquet F. (2019). New protein-DNA complexes in archaea: A small monomeric protein induces a sharp V-turn DNA structure. Sci. Rep..

[84] Hocher A., Rojec M., Swadling J.B., Esin A., Warnecke T. (2019). The DNA-binding protein HTa from *Thermoplasma acidophilum* is an archaeal histone analog. eLife.

[85] Bai L., Xie T., Hu Q., Deng C., Zheng R., Chen W. (2015). Genome-wide comparison of ferritin family from Archaea, Bacteria, Eukarya, and Viruses: Its distribution, characteristic motif, and phylogenetic relationship. Naturwissenschaften.

[86] Jones D.L., Baxter B.K. (2017). DNA repair and photoprotection: Mechanisms of overcoming environmental ultraviolet radiation exposure in halophilic archaea. Front. Microbiol..

[87] Marshall C.J., Santangelo T.J. (2020). Archaeal DNA repair mechanisms. Biomolecules.

[88] Setlow P. (2014). Spore resistance properties. Microbiol. Spectr..

[89] Yang L., Li L. (2015). Spore photoproduct lyase: The known, the controversial, and the unknown. J. Biol. Chem..

[90] Djouiai B., Thwaite J.E., Laws T.R., Commichau F.M., Setlow B., Setlow P., Moeller R. (2018). Role of DNA repair and protective components in *Bacillus subtilis* spore resistance to inactivation by 400-nm-wavelength blue light. Appl. Environ. Microbiol..

[91] Lessa F.C., Mu Y., Bamberg W.M., Beldavs Z.G., Dumyati G.K., Dunn J.R., Farley M.M., Holzbauer S.M., Meek J.I., Phipps E.C. (2015). Burden of *Clostridium difficile* infection in the United States. N. Engl. J. Med..

[92] Smits W.K., Lyras D., Lacy D.B., Wilcox M.H., Kuijper E.J. (2016). *Clostridium difficile* infection. Nat. Rev. Dis. Primers.

[93] Centers for Disease Control and Prevention (2012). Bioterrorism Agents/Diseases A to Z by Category: Category A.

[94] Talukdar P.K., Olguín-Araneda V., Alnoman M., Paredes-Sabja D., Sarker M.R. (2015). Updates on the sporulation process in *Clostridium* species. Res. Microbiol..

[95] Hoch J.A. (1993). Regulation of the phosphorelay and the initiation of sporulation in *Bacillus subtilis*. Annu. Rev. Microbiol..

[96] Setlow P. (2006). Spores of *Bacillus subtilis*: Their resistance to and killing by radiation, heat and chemicals. J. Appl. Microbiol..

[97] Lee K.S., Bumbaca D., Kosman J., Setlow P., Jedrzejas M.J. (2008). Structure of a protein–DNA complex essential for DNA protection in spores of *Bacillus* species. Proc. Natl. Acad. Sci. USA.

[98] Setlow P. (1995). Mechanisms for the prevention of damage to DNA in spores of *Bacillus* species. Annu. Rev. Microbiol..

[99] Tennen R., Setlow B., Davis K.L., Loshon C.A., Setlow P. (2000). Mechanisms of killing of spores of *Bacillus subtilis* by iodine, glutaraldehyde and nitrous acid. J. Appl. Microbiol..

[100] Meaney C.A., Cartman S.T., McClure P.J., Minton N.P. (2016). The role of small acid-soluble proteins (SASPs) in protection of spores of *Clostridium botulinum* against nitrous acid. Int. J. Food Microbiol..

[101] Raju D., Waters M., Setlow P., Sarker M.R. (2006). Investigating the role of small, acid-soluble spore proteins (SASPs) in the resistance of *Clostridium perfringens* spores to heat. BMC Microbiol..

[102] Raju D., Setlow P., Sarker M.R. (2007). Antisense-RNA-mediated decreased synthesis of small, acid-soluble spore proteins leads to decreased resistance of *Clostridium perfringens* spores to moist heat and UV radiation. Appl. Environ. Microbiol..

[103] Li J., McClane B.A. (2008). A novel small acid soluble protein variant is important for spore resistance of most *Clostridium perfringens* food poisoning isolates. PLoS Pathog..

[104] Li J., Paredes-Sabja D., Sarker M.R., McClane B.A. (2009). Further characterization of *Clostridium perfringens* small acid soluble protein-4 (Ssp4) properties and expression. PLoS ONE.

[105] Paredes-Sabja D., Raju D., Torres J.A., Sarker M.R. (2008). Role of small, acid-soluble spore proteins in the resistance of *Clostridium perfringens* spores to chemicals. Int. J. Food Microbiol..

[106] Nerber H.N., Sorg J.A. (2021). The small acid-soluble proteins of *Clostridioides difficile* are important for UV resistance and serve as a check point for sporulation. PLoS Pathog..

[107] Greipel J., Urbanke C., Maass G., Saenger W., Heinemann U. (1989). The single-stranded DNA binding protein of *Escherichia coli*: Physicochemical properties and biological functions. Protein-Nucleic Acid Interaction.

[108] Shereda R.D., Kozlov A.G., Lohman T.M., Cox M.M., Keck J.L. (2009). SSB as an organizer/mobilizer of genome maintenance complexes. Crit. Rev. Biochem. Mol. Biol..

[109] Oliveira M.T., Ciesielski G.L., Oliveira M.T. (2021). The essential, ubiquitous single-stranded DNA-binding proteins. Single Stranded DNA Binding Proteins.

[110] Raghunathan S., Ricard C.S., Lohman T.M., Waksman G. (1997). Crystal structure of the homo-tetrameric DNA binding domain of *Escherichia coli* single-stranded DNA-binding protein determined by multiwavelength x-ray diffraction on the selenomethionyl protein at 2.9-Å resolution. Proc. Natl. Acad. Sci. USA.

[111] Filipkowski P., Koziatek M., Kur J. (2006). A highly thermostable, homodimeric single-stranded DNA-binding protein from *Deinococcus radiopugnans*. Extremophiles.

[112] Bernstein D.A., Eggington J.M., Killoran M.P., Misic A.M., Cox M.M., Keck J.L. (2004). Crystal structure of the *Deinococcus radiodurans* single-stranded DNA-binding protein suggests a mechanism for coping with DNA damage. Proc. Natl. Acad. Sci. USA.

[113] Lockhart J.S., DeVeaux L.C. (2013). The essential role of the *Deinococcus radiodurans* ssb gene in cell survival and radiation tolerance. PLoS ONE.

[114] George N.P., Ngo K.V., Chitteni-Pattu S., Norais C.A., Battista J.R., Cox M.M., Keck J.L. (2012). Structure and cellular dynamics of *Deinococcus radiodurans* single-stranded DNA (ssDNA)-binding protein (SSB)-DNA complexes. J. Biol. Chem..

[115] Zhang J., Zhou R., Inoue J., Mikawa T., Ha T. (2014). Single molecule analysis of *Thermus thermophilus* SSB protein dynamics on single-stranded DNA. Nucleic Acids Res..

